# Developing an ICD-10 Coding Assistant: Pilot Study Using RoBERTa and GPT-4 for Term Extraction and Description-Based Code Selection

**DOI:** 10.2196/60095

**Published:** 2025-02-11

**Authors:** Sander Puts, Catharina M L Zegers, Andre Dekker, Iñigo Bermejo

**Affiliations:** 1 Department of Radiation Oncology (Maastro), GROW Research Institute for Oncology and Reproduction, Maastricht University Medical Centre+, P.O. Box 616, Maastricht, 6200 MD, Netherlands, 31 43 38 81863; 2 Data Science Institute (DSI), Hasselt University, Belgium

**Keywords:** International Classification of Diseases, ICD-10, computer-assisted-coding, GPT-4, coding, term extraction, code analysis, computer assisted coding, transformer model, artificial intelligence, AI automation, retrieval-augmented generation, RAG, large language model, LLM, Bidirectional Encoder Representations from Transformers, Robustly Optimized BERT Pretraining Approach, RoBERTa, named entity recognition, NER

## Abstract

**Background:**

The International Classification of Diseases (ICD), developed by the World Health Organization, standardizes health condition coding to support health care policy, research, and billing, but artificial intelligence automation, while promising, still underperforms compared with human accuracy and lacks the explainability needed for adoption in medical settings.

**Objective:**

The potential of large language models for assisting medical coders in the ICD-10 coding was explored through the development of a computer-assisted coding system. This study aimed to augment human coding by initially identifying lead terms and using retrieval-augmented generation (RAG)–based methods for computer-assisted coding enhancement.

**Methods:**

The explainability dataset from the CodiEsp challenge (CodiEsp-X) was used, featuring 1000 Spanish clinical cases annotated with ICD-10 codes. A new dataset, CodiEsp-X-lead, was generated using GPT-4 to replace full-textual evidence annotations with lead term annotations. A Robustly Optimized BERT (Bidirectional Encoder Representations from Transformers) Pretraining Approach transformer model was fine-tuned for named entity recognition to extract lead terms. GPT-4 was subsequently employed to generate code descriptions from the extracted textual evidence. Using a RAG approach, ICD codes were assigned to the lead terms by querying a vector database of ICD code descriptions with OpenAI’s text-embedding-ada-002 model.

**Results:**

The fine-tuned Robustly Optimized BERT Pretraining Approach achieved an overall *F*_1_-score of 0.80 for ICD lead term extraction on the new CodiEsp-X-lead dataset. GPT-4-generated code descriptions reduced retrieval failures in the RAG approach by approximately 5% for both diagnoses and procedures. However, the overall explainability *F*_1_-score for the CodiEsp-X task was limited to 0.305, significantly lower than the state-of-the-art *F*_1_-score of 0.633. The diminished performance was partly due to the reliance on code descriptions, as some ICD codes lacked descriptions, and the approach did not fully align with the medical coder’s workflow.

**Conclusions:**

While lead term extraction showed promising results, the subsequent RAG-based code assignment using GPT-4 and code descriptions was less effective. Future research should focus on refining the approach to more closely mimic the medical coder’s workflow, potentially integrating the alphabetic index and official coding guidelines, rather than relying solely on code descriptions. This alignment may enhance system accuracy and better support medical coders in practice.

## Introduction

### Background and Significance

The International Classification of Diseases (ICD), developed by the World Health Organization (WHO), serves as a universal standard for coding health-related conditions [1]. The ICD system steers health care policymaking, assists in billing and reimbursement, underpins research, ensures quality monitoring, and promotes the standardization of medical information exchange. ICD-10 is divided into diagnosis and procedure codes, encompassing over 100,000 codes. The system is hierarchically organized and includes detailed coding guidelines.

Medical coding entails the allocation of unique codes to medical records and is a standard procedure in most hospitals. Hospitals collect a list of ICD codes relevant to each patient’s hospital admission. Medical coding is a time-consuming and error-prone task, which has led to interest in automating it, and in turn, to the emergence of the medical coding subfield within medical natural language processing (NLP) [1,2]. However, the task of effectively computerizing ICD coding remains a challenge. While the accuracy of codes assigned by medical coders may be debated [2-4], current state-of-the-art (SOTA) automated systems for ICD-10 coding still fall short of human performance [5-7]. In addition, many artificial intelligence (AI) solutions are “black boxes,” not offering explanations for their predictions. However, model transparency is required to gain trust, which is crucial for its adoption in medical settings [8].

### ICD Coding

Diagnosis codes in the ICD system are organized into an alphanumeric format across 21 chapters, capturing a range of categories including specific diseases, symptoms, and external causes, among others.

**Figure 1.**
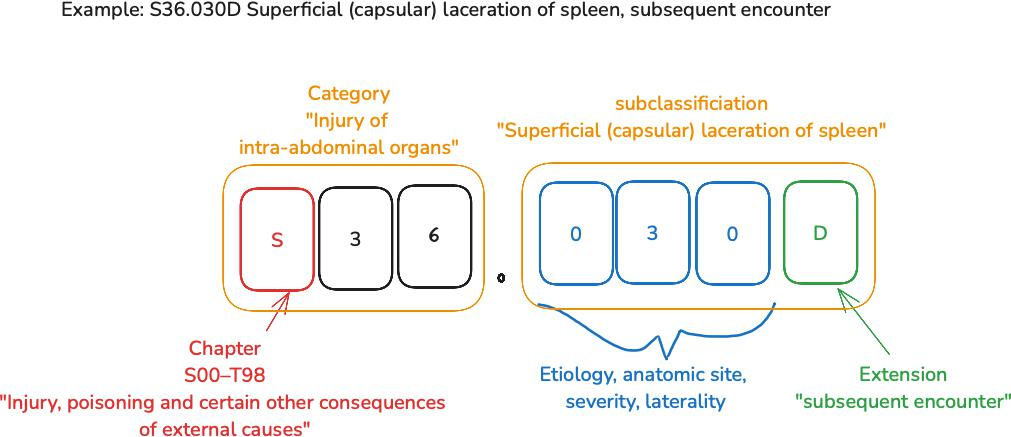
Structure and components of an ICD-10 diagnosis code. ICD-10: International Classification of Diseases, 10th version.

**Figure 2.**
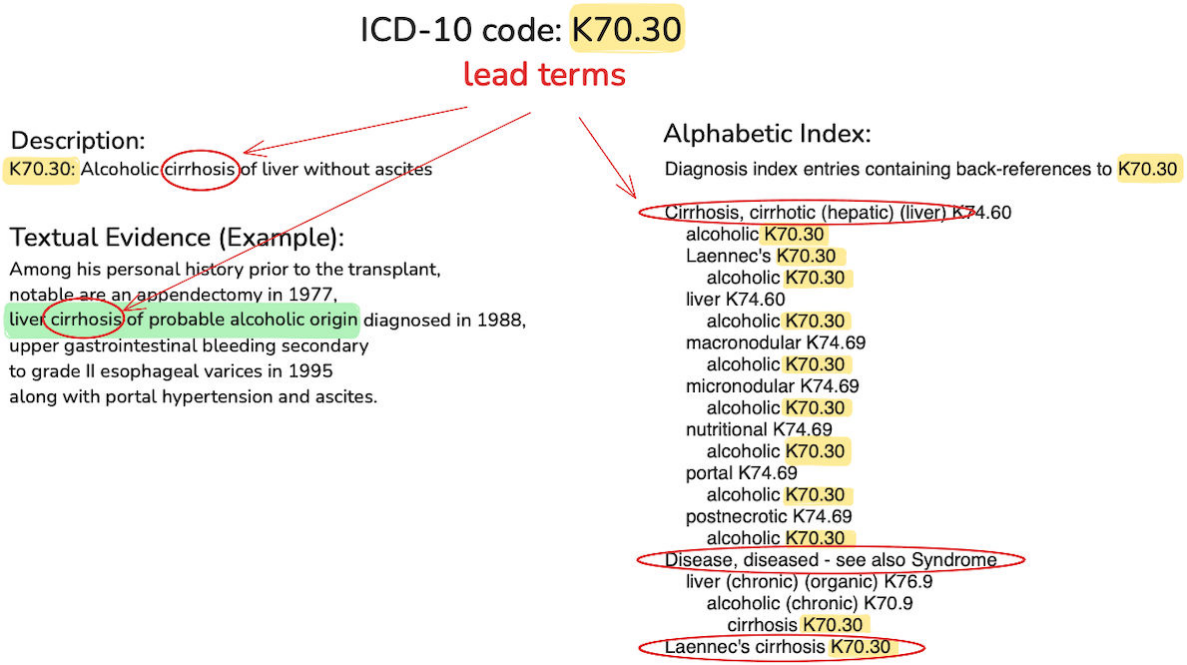
Illustration of concepts “code description,” “textual evidence (green),” “lead terms (red),” and “back references” in the “alphabetical index”. ICD: International Classification of Diseases.

The hierarchical structure of ICD codes begins with a letter that corresponds to a chapter, together with the 2 subsequent digits that form the category of the code. Following the category code, the subclassification uses up to 5 additional alphanumeric characters. These detail specific aspects of the disease or condition, like laterality or severity, as illustrated in Figure 1. The procedure codes, separate from diagnostic codes, consist of 7 characters termed as “axis,” with each axis representing a specific aspect of the medical procedure performed [9]. ICD-10 provides a comprehensive 3-volume coding manual that includes an alphabetical index. This index helps in finding medical codes using “lead terms,” which are crucial keywords associated with medical conditions, listed in the alphabetical index. Medical coders first identify a diagnosis’s lead term in the text (Figure 2). In this example, the lead term “cirrhosis” from the textual evidence corresponds to an alphabetic index entry. Under “Cirrhosis,” the relevant subentry is “liver,” “alcoholic,” leading to the back-reference code K70.30. The next coding step involves consulting the tabular index for K70.30 for detailed instructions.

For each diagnosis and procedure that needs an ICD code, the initial step is to identify the lead term, which is comparable to the named entity recognition (NER) task in NLP. NER involves extracting specific categories like names, locations, medications, and diseases from unstructured text. Only a few studies proposing lead term-based approaches for ICD coding have been published [5], possibly due to the absence of public datasets annotated with lead terms. Previous NER approaches focused on extracting full textual evidence, rather than lead terms [5].

### Explainability

Recent studies have proposed adversarial robustness training strategies and feature attribution, such as the use of attention mechanisms, for the explainability of NLP [10-12]. A limitation of these approaches is the absence of efforts to validate whether the identified “explainable” terms align with official ICD guidelines. For instance, while “insulin” may be a predictive term for the disease “diabetes”, it is not suitable as evidence for coding according to the WHO guidelines and educational textbooks [13] as insulin can be prescribed for various medical conditions other than diabetes.

### Transformers

In the original transformer architecture, which encompasses both encoder and decoder components [14], it is crucial to recognize the distinct roles of these elements. Encoder-only models, such as the BERT (Bidirectional Encoder Representations from Transformers) architecture and its variant, RoBERTa (Robustly Optimized BERT Pretraining Approach) [15], excel at capturing relevant information from the input data and constructing meaningful representations [16-18] and can be found in current SOTA models in ICD coding [5,7]. Conversely, decoder models, exemplified by GPT, are specialized in generating coherent and contextually relevant text [19]. With the evolution of the GPT architecture, remarkable advancements have been achieved in NLP [20,21]. GPT has already been applied across various health care disciplines and use cases [22]. However, early studies indicate that while GPT-3.5, GPT-4, and ChatGPT show potential, their performance in ICD classification has been mixed, with out-of-the-box negative results [23]. Generative approaches may be especially useful for ICD coding of rare diseases [24]. OpenAI’s GPT was, for a time, the leading large language model (LLM) in generative AI [20], but other models, such as Mixtral, Gemini, Grok, Llama, and Claude [25], are available and quickly catching up [26,27].

### Objective

Our objective was to develop a GPT-4-based computer-assisted coding system to aid medical coders in ICD-10 coding. Our approach enhances the identification of diagnoses and procedures in texts by initially focusing on lead terms rather than extracting the full textual evidence for a code at once. The reason for initially extracting lead terms is that it aligns perfectly with the first step followed by medical coders in their procedure. This approach would therefore be a good fit for assisting with the process. For code normalization, our approach exclusively employs code descriptions, moving away from reliance on model-based training. Specifically, the ICD code for identified lead terms was assigned using GPT-4 and ICD descriptions, employing a retrieval-augmented generation (RAG) approach. This reliance on an external knowledge source is strategically chosen to improve generalizability, enhancing the system’s ability to accurately classify codes unseen during training. Furthermore, this method offers increased adaptability to changes in the coding system. The research also investigates the optimal method for querying a database to retrieve ICD codes by description. It compares the effectiveness of 2 approaches: using direct textual evidence as the query and employing code descriptions generated (invented) by GPT-4 from that textual evidence. Additionally, the study aimed to assess GPT-4’s ability to accurately select the best code by matching code descriptions with textual diagnoses. This objective is rooted in the understanding that ICD coding guidelines and descriptions are designed for humans, and it seeks to determine if GPT-4’s capabilities in following instructions translate to robust performance in this specific context.

## Methods

### GPT-4

This study used GPT-4 (gpt-4 1106-preview) via OpenAI’s application programming interface and the Langchain Python library [11]. To ensure reproducibility, the model’s temperature was set to 0. GPT-4’s parametric knowledge, or out-of-the-box performance, was evaluated in 2 ways. First, it was prompted to generate descriptions for 100 random ICD codes from the CodiEsp dataset. These descriptions were then compared with the ground truth by prompting GPT-4 in a new chat with, “Do both descriptions refer to the same ICD code?” Second, GPT-4 was tasked with matching the correct ICD-10 code to each of the 100 official descriptions.

### Dataset CodiEsp

CodiEsp is a unique corpus of 1000 clinical cases in Spanish, annotated by professionals for explainability, offering token-level annotations that provide textual evidence for each assigned code [28]. The annotation agreement rates were 88.6% for diagnosis, 88.9% for procedures, and 80.5% for textual evidence [28]. The test set contains 1514 unique ICD codes (1143 diagnoses and 371 procedures), some of which are not present in the training set, specifically, 439 diagnosis codes (38.3%) and 231 procedure codes (62.3%). Among the procedure codes, 143 (38.5%) are 4-axis ICD-10 procedure codes, which officially should have 7-axis ICD-10 codes. These incomplete codes lack descriptions and account for 602 of the total of 1112 procedure codes (54%). Overall, 828 out of 4777 (17.3%) test annotations do not have an exact match in the training set. In the current SOTA on CodiEsp-X, the entity extraction and normalization are both transformer-based [5].

### Dataset CodiEsp-X-Lead

This approach does not use NER to extract complete textual evidence; instead, it mimics human coders by first identifying lead terms. An annotated dataset was required to train and evaluate a NER model for this task. However, manual annotation of 18,435 items was deemed infeasible due to the time and effort involved. Instead, GPT-4 few-shot prompting with 20 manually curated examples was used to extract lead terms from CodiEsp-X textual evidence. The author (SP) reviewed and corrected the extraction. The resulting CodiEsp-X-lead dataset is identical to CodiEsp-X but includes additional columns for the GPT-extracted lead terms.

### Approach

**Figure 3.**
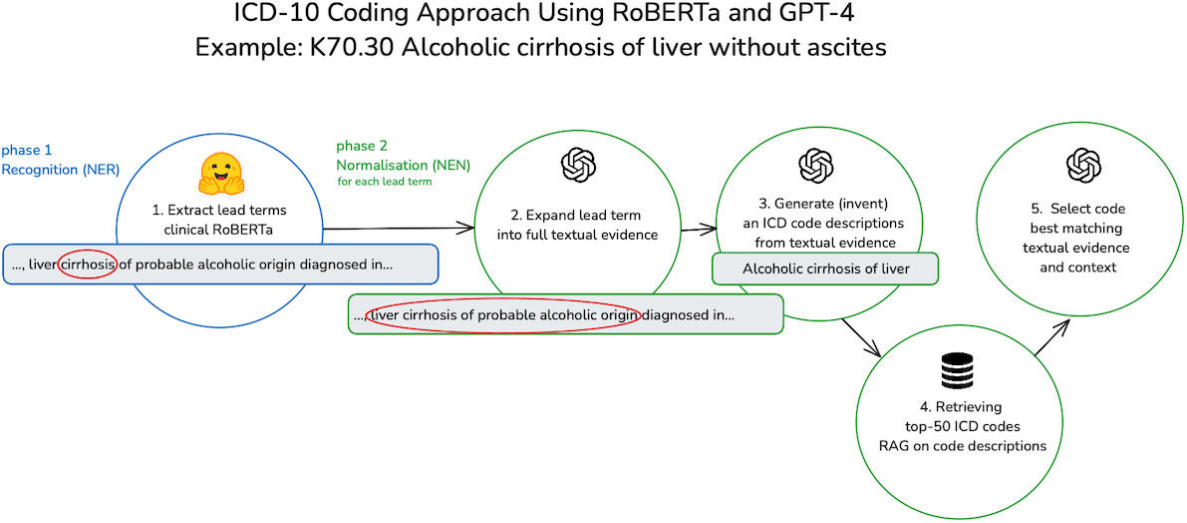
Schematic overview of the process followed for ICD coding. ICD: International Classification of Diseases; RAG: retrieval-augmented generation; RoBERTa: Robustly Optimized BERT Pretraining Approach

For this study, we followed the process described in 2 main phases and 5 steps in Figure 3, mimicking the process human medical coders follow.

#### Step 1: Extract Lead Terms

A RoBERTa-based biomedical-clinical language model for Spanish was fine-tuned for NER of lead terms using the CodiEsp-X-lead dataset and Hugging Face’s transformer library (version 4.26.0) [19,20], using the official dataset splits. The fine-tuned model enabled precise identification of lead terms within medical documents. To evaluate its performance, the same approach was applied to the CodiEsp-X dataset to extract full-textual evidence, which is more commonly used in AI methods for code assignment.

#### Step 2: Expand Lead Terms Into Full Textual Evidence

Instead of using NER to extract textual evidence, GPT-4 was instructed to complete a template for each lead term which included a field to capture the full textual evidence for the code. Alongside the textual evidence, the template includes a field designed to capture contextual information, which is described as the text snippet containing all the necessary information for coding the lead term. In the prompt, the entire text was supplied, using HTML tags to highlight the lead terms within the original text.

#### Step 3: Generate (Invent) an ICD Code Description From Textual Evidence

In this part, we tested the theory that GPT-4 can improve text embeddings-based database search accuracy by aligning queries with official ICD code descriptions. This assumption arose from the observation that terms in official ICD code descriptions often markedly differ from those used in clinical texts. GPT-4 was tasked to generate (invent) ICD code descriptions from extracted textual evidence, aiming to reconcile these terminological discrepancies. This process, known as query alignment, refines a query to mirror the language and structure of database entries more closely, with the goal of enhancing search accuracy. To verify this assumption, the study compared different types of input queries; raw clinical text versus GPT-4 refined descriptions, to assess their effectiveness in achieving precise database lookups.

#### Step 4: Retrieving ICD Codes Using Descriptions and RAG

The complete lists of code descriptions for diagnoses and procedures contain around 2.5 million tokens each, far exceeding the context window size of current generative models. While including an entire knowledge base in the prompt is feasible for smaller datasets, RAG is necessary here to retrieve relevant context efficiently. To address this, we converted the official ICD code descriptions into vectors using OpenAI’s “text-embedding-ada-002” model via the Langchain library [21], and stored them in the Chroma vector database. This enables efficient searching by embedding-based similarity rather than exact matches. By retrieving only the most relevant information, RAG ensures that the results fit within the model’s context window, allowing it to process the required details effectively.

#### Step 5: Select the Code Best Matching Textual Evidence

From the 50 results retrieved from the vector store query, GPT-4 was prompted to choose the best-matching ICD code. For the decision, GPT was provided with the lead term, textual evidence, and additional context extracted in Step 2.

### Evaluation

The performance of the ICD coding pipeline and its components was evaluated using precision, recall, and *F*_1_-score metrics calculated with the CodiEsp scripts [18,22]. To assess performance loss at each step, predictions were replaced with actual values in subsequent steps only if the predictions at the current step were accurate. Specifically, in lead term extraction, a prediction was deemed correct if the predicted text span overlapped with the textual evidence. For textual evidence extraction, if there was an overlap, all values except the span were replaced. In the code retrieval step, the predicted code was replaced with the actual code if the predicted code was among the top-k results. In the code assignment, the predictions were not altered. Additionally, the accuracy of lead term extraction was also measured using *F*_1_-scores calculated for the actual spans, independently of the CodiEsp script.

### Ethical Considerations

This study used only publicly available data from the CodiEsp challenge, a synthetic corpus of 1000 clinical case studies, that does not contain real patient information, ensuring anonymity and eliminating the need for ethical approval.

## Results

### GPT-4

GPT-4 generated descriptions for 100 randomly selected ICD diagnosis codes and in a new chat compared these with official ICD-10 descriptions. It found that only 52% matched the official descriptions, as detailed in Multimedia Appendix 1. When GPT-4 was tasked with associating the correct ICD-10 code to each of the 100 official descriptions, only 47% of the ICD codes were correct.

### Dataset CodiEsp-X-Lead

A key outcome of this study is the creation of the CodiEsp-X-lead dataset using GPT-4. This dataset consists of 7748 unique textual evidence strings, with 6532 of these containing multiple terms. As instructed, GPT-4 typically extracted a single lead term from these multiterm strings. However, in 549 cases (8%), multiple lead terms were extracted. The researchers manually resolved 325 of these cases to a single lead term, leaving the remaining cases unmapped due to ambiguity in determining the appropriate lead term.

### Step 1: Extracting Lead Terms

After fine-tuning the Biomedical-clinical language model for Spanish for NER to extract lead terms [29], it achieved an *F*_1_-score of 0.80 (precision of 0.78 and a recall) of 0.83 on the CodiEsp-X-lead test set (Table 1). When this model was solely fine-tuned and tested for diagnoses, it exhibited a slightly better performance, with an *F*_1_-score of 0.82 (Table 1).

**Table 1.** Named entity recognition (NER) performance on extracting lead terms and textual evidence for diagnoses and or procedures on CodiEsp-X and CodiEsp-X-lead.

NER	*F*_1_-score
Textual evidence diagnoses and procedures combined (ours-baseline) [29]	0.65
Textual evidence diagnoses (SOTA^a^) [5]	0.71
Textual evidence procedures (SOTA) [5]	0.61
Main-terms diagnoses (ours) [29]	0.82
Main-terms diagnoses and procedures combined (ours) [29]	0.80

a SOTA: state-of-the-art.

### Step 2: Expand Lead Terms Into Full Textual Evidence

**Figure 4.**
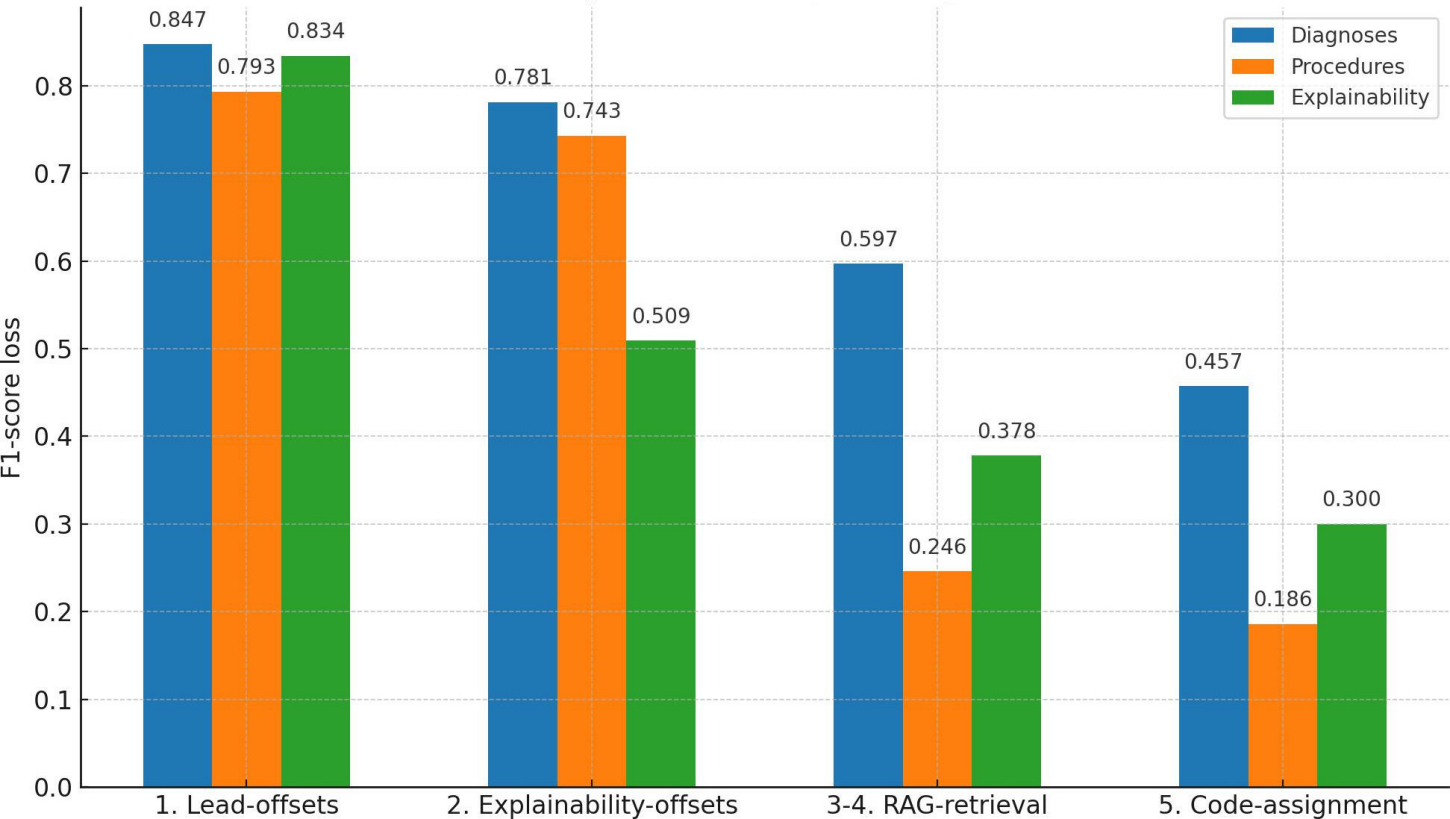
F1-Score performance loss for each individual step in the approach.

Using GPT-4, full textual evidence was extracted based on preannotated lead terms, which were identified in Step 1. GPT-4 completed a template that incorporated the full textual evidence for each provided lead term. This approach achieved an *F*_1_-score of 0.509, as shown in Figure 4.

**Figure 5.**
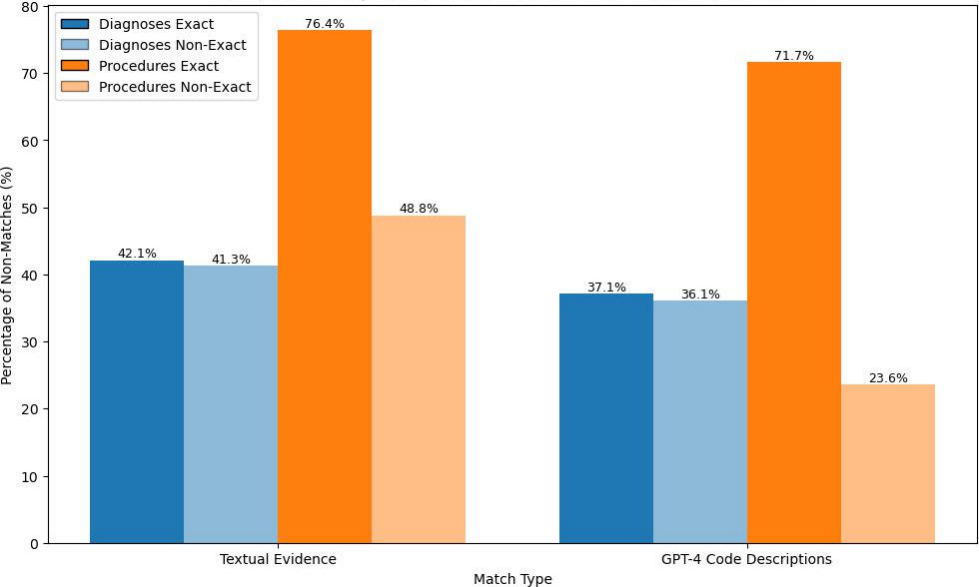
Comparison of failed retrievals @50 in a vector database containing all official ICD diagnosis and procedure codes. The chart compares results for 25 documents (507 codes), contrasting searching by textual evidence versus GPT-4 zero-shot generated code descriptions based on the textual evidence.

The base model from Step 1 was fine-tuned to extract textual evidence solely for benchmarking, achieving an *F*_1_-score of 0.65 on the CodiEsp-X test set (Table 1). This model was not used in the actual approach, which aims to mimic medical coders by prioritizing the extraction of lead terms. Note that results from Table 1, assessing exact offsets, differ from those in Figure 5 where the official CodiEsp scripts allow for an error margin.

### Step 3: Generate (Invent) an ICD Code Description From Textual Evidence

GPT-4 was used to generate ICD code descriptions from textual evidence, evaluated on 25 documents containing 507 codes in a RAG setting. When comparing GPT-4-generated descriptions to direct retrieval using textual evidence (Figure 5), retrieval failures decreased from 160 to 141 (42.1% to 37.1%) for the 380 diagnoses codes and from 97 to 91 (76.4% to 71.7%) for the 127 procedure codes.

For procedure codes, many were assigned as 4-axis codes, lacking the detail of the official 7-axis codes and without available descriptions in the resources. As a comparison, a nonexact matching criterion was applied, where a match was considered valid if the assigned code was a substring of the retrieved code. Under this nonexact approach, retrieval failures for procedures dropped significantly, from 62 to 30 out of 127 (48.8% to 23.6%). For diagnoses, which were mostly assigned in full detail, the decrease in failures was smaller, from 157 to 137 out of 380 (41.3% to 36.1%).

### Step 4: Retrieving ICD Codes Using Descriptions and RAG

When RAG retrieval was evaluated within the full pipeline, a significant drop in explainability *F*_1_-score was observed, falling from 0.509 to 0.378, as shown in steps 3 and 4 in Figure 4. Procedures had the largest contribution to this drop, with their *F*_1_-score decreasing from 0.743 to 0.246. Diagnoses also experienced a decline, with their *F*_1_-score dropping from 0.781 to 0.597.

### Step 5: Select the Code Best Matching Textual Evidence

In the final step, GPT-4 had to select the correct ICD code from the top 50 retrieved results based on the previously predicted textual evidence. This selection process caused a further decline in performance, with the explainability *F*_1_-score dropping from 0.378 to 0.3 (Table 2 and Figure 4).

**Table 2.** Comparison of performance of different models in the CodiEsp challenge.

Team name	Diagnoses(*F*_1_-score)	Procedures(*F*_1_-score)	Explainability (*F*_1_-score)
FLE	0.679	0.514	0.611
IAM	0.687	0.522	0.611
BETO-Galén+mBERTGalén+XLM-R-Galén	—^a^	—	0**.**633
Ours	0.457	0.204	0.305

a Not available.

## Discussion

### Summary of Main Findings

In this study, we proposed methods to assist ICD-10 coding using LLMs. We successfully created a dataset of lead terms, “CodiEsp-X-lead,” using few-shot prompting on GPT-4. Using this dataset, we effectively fine-tuned a transformer model (*F*_1_-score of 0.80) that can assist medical coders in the initial step of medical coding: identifying ICD lead terms. To enhance RAG retrieval, we employed GPT-4 to generate code descriptions from textual evidence, aiming to improve ICD code identification within a code description database. Although the initial step, focusing on extracting lead terms, closely mimicked medical coders and performed well, subsequent RAG steps based on textual descriptions were less effective, yielding a poor *F*_1_-score of 0.305 on the CodiEsp-X task. We will discuss the shortcomings of the RAG implementation and analyze each step’s performance, highlighting areas of significant improvement.

### Motivation of Choices

RoBERTa was selected over GPT-4 for NER of lead terms because encoder-based models are better suited for token-level tasks like NER, providing detailed context understanding. RoBERTa is lighter and more efficient to fine-tune, potentially outperforming generative, decoder-only models like GPT-4 [17,30]. Additionally, as of 2023, fine-tuning GPT-4 was not possible. Previous research indicates that GPT models perform poorly in traditional, example-rich NER tasks [31].

In contrast, GPT-4 excels in few-shot NER tasks and was chosen for other aspects of the study due to its SOTA performance in NLP. Medical coding involves following specific guidelines, and a well-configured generative model like GPT-4, trained for instruction-following, is likely to perform well in such roles. Therefore, GPT-4 was used to create the dataset by extracting lead terms through few-shot NER, as manually compiling the dataset was not feasible. Its natural language generation capabilities and instruction-following skills were also leveraged to generate code descriptions, align queries, and identify the correct ICD-10 codes from a given list.

Using code descriptions exclusively for normalizing lead terms to ICD codes offers independence from training data-based fine-tuning. Given the vastness of ICD-10, with over 100,000 codes, many are rarely encountered in practice. The analysis of the CodiEsp dataset revealed that 17.3% of specific test set codes were not included in the training data. If the proposed method, which primarily relies on official code descriptions, were to be successful, it likely would be very generalizable across different ICD datasets.

### GPT-4 Parameteric Knowledge

The preliminary results of GPT-4’s performance were disappointing. However, it is crucial to approach negative outcomes based on prompts with caution, as the prompts themselves may be suboptimal. Nevertheless, we can definitively state that GPT-4’s out-of-the-box performance on 2 basic ICD-10 coding tasks—coding official descriptions and generating code descriptions for diagnoses—was mediocre. The approach of using GPT-4 generated descriptions for procedure codes was found to be completely ineffective in the study. These findings and those from the first available publications on GPT-3.5 and GPT-4’s abilities with ICD billing codes underscore their subpar performance for downstream applications [23].

### Creating CodiEsp-X-Lead With GPT-4

In this study, we introduced “CodiEsp-X-lead”, a curated selection of lead terms from the CodiEsp-X dataset. Using GPT-4 as an annotator and employing few-shot prompting, we extracted these terms from the dataset’s textual evidence. This method showcases its utility for other use cases where manual annotation is unfeasible, but reviewing the generated annotations is feasible. While the authors manually reviewed and made minor adjustments to this extraction, there is potential for further refinement. For improved lead term identification, lead terms derived from the codes assigned to that textual evidence could be suggested by using an inverse lookup on the official alphabetical index. This approach can benefit both the GPT-4 prompts given and our manual review process. Last, to ensure the highest accuracy, domain experts, such as medical coders, could annotate or validate a portion of the lead terms and can help improve the quality of the examples used in the (few-shot) prompt. Another suggestion to enhance the dataset’s quality is to allow multiple lead terms for each assigned code. Limiting to a single lead term when several are applicable results in inconsistencies and reduces the quality of the dataset and the model’s performance.

### Step 1: Extracting Lead Terms

The results demonstrate that using NER for lead term identification holds distinct advantages over extracting the full textual evidence all at once.

First, since the task is more straightforward, significantly better performance can be obtained when extracting lead terms compared with extracting the full textual evidence. For instance, the SOTA approach for the CodiEsp-X task yielded an *F*_1_-score of 0.71 for NER of full-textual evidence for diagnoses compared with our score of 0.82 for NER of lead terms (Table 1) [2]. High performance in extracting the correct lead term is crucial, as it sets the performance ceiling for the entire coding approach.

Second, starting with lead term extraction mirrors the initial step of human coders, facilitating seamless integration into their workflow and providing standalone value. A coding assistant that accurately identifies lead terms with high recall and precision would likely be favored over a system that offers full ICD assignments but has a higher error rate. The latter could compel human coders to frequently reread the text and make extensive corrections, frequently starting from scratch. In contrast, a fully autonomous approach that meets performance requirements without needing human intervention would likely favor extracting full-textual evidence at once, due to its potential for higher efficiency. However, in such a scenario, the relevance of explainability becomes questionable, particularly when the system operates satisfactorily with no human oversight.

The proposed NER model for extracting lead terms performs well, but further improvements can be achieved by enhancing the quality of the CodiEsp-X-lead dataset. It may be beneficial to fine-tune the model to prioritize recall over precision, as medical coders might favor removing incorrect lead term suggestions instead of revisiting a text to verify that all diagnoses are captured. Additionally, optimizing parameters might enhance the performance of the model.

### Step 2: Expand Lead Terms Into Full Textual Evidence

From Step 2, our method deviated from ICD-10 coding guidelines by adopting a RAG approach, which requires a database lookup rather than following the traditional coding steps.

Using a zero-shot GPT-4 prompt to extract full textual evidence resulted in mediocre outcomes, with the *F*_1_-score for explainability dropping from 0.824 to 0.509 (Figure 4). This highlights a significant limitation of this approach. In contrast, skipping lead term extraction and directly employing a fine-tuned BERT model yielded better performance, achieving *F*_1_-scores of 0.71 and 0.61 for diagnoses and procedures respectively, which are SOTA, and a combined score of 0.65 for our model [5]. For future work, we propose to align more closely with medical coders’ practices by continuing to use extracted lead terms and leveraging the alphabetic index and official guidelines for code lookups, allowing textual evidence to emerge naturally. This differs from the current RAG approach, which relies on a single lookup using predefined semantic queries.

### Step 3: Generate (Invent) an ICD Code Description From Textual Evidence

The hypothesis we examined suggested that GPT-generated code descriptions, based on textual evidence and context, would semantically more closely align with a database of ICD code descriptions than the original textual evidence. This expected alignment is intended to enhance RAG retrieval, suggesting that rephrasing textual evidence into descriptions using AI could improve the accuracy of searching for ICD codes by their descriptions. From the experiments on a subset of the data (Figure 5), we confirm the hypothesis, as all cases show a reduction in failed retrievals. Although the improvements are subtle in most scenarios, with only a few percentage points fewer failed retrievals, there is a marked improvement for nonexact matching procedure codes. Specifically, failed retrievals dropped from 48.8% to 23.6%. This notable performance in nonexact matching is likely due to the majority of procedure codes lacking descriptions (59%), which is why nonexact matching was evaluated and resulted in a significant reduction in the number of classes.

### Step 4: Retrieving ICD Codes Using Descriptions and RAG

In step 3, we tested nonexact matching for procedure codes, but the official Codiesp evaluation uses exact matching. Our approach, relying on NER extraction and descriptions for code lookup, fails when many assigned codes lack descriptions and are invalid.

In the RAG retrieval step, *F*_1_-score performance for procedure codes drops sharply from 0.743 to 0.246. Diagnosis code performance is also mediocre at 0.597, but this would still be acceptable if GPT-4 could select the correct code in the next step without causing another drop.

### Step 5: Select the Code Best Matching Textual Evidence

In step 5, GPT-4 selects the final answer from the retrieved codes, but another drop in performance is observed, resulting in a final *F*_1_-score of 0.3 on CodiEsp-x, compared with the SOTA score of 0.633 (Table 2) [5]. It is likely that the proposed textual evidence (span) is incorrect in some cases, making it valuable to evaluate this step in isolation to assess its impact on overall performance.

### Method Recap and Evaluation

While the initial NER step for extracting lead terms is promising and can directly assist medical coders, subsequent steps perform inadequately. Although using RAG with code descriptions seems intuitive for ICD-10 coding, the implementation underperforms. While lead term extraction works well, the following steps should better mimic the workflow of medical coders. If this is not done, step 2 could be replaced by extracting textual evidence via NER, which achieves a higher *F*_1_-score (0.65 in Table 1) compared with the current method (0.509 in Figure 5).

Instead of relying on descriptions to find the correct code, it would be more effective to use the alphabetic index of lead terms. Textual evidence could then be derived from this lookup using iterative steps, rather than the current approach, which requires knowing the exact spans of evidence in advance before performing the database lookup, as we need to know exactly what we are searching for.

Additionally, terms like NEC (not elsewhere classified) and NOS (not otherwise specified), which are present in some code descriptions, could be useful for an agent-based approach, allowing an agent to interpret and follow these instructions in coding scenarios. This would enhance performance, as an agent can adaptively handle these cases, which are often challenging for a simple database lookup.

Incorporating more coding guidelines into the process would further align with an agent-based model, closely replicating the real-world workflow of medical coders.

### Limitations

Our hypothesis was that GPT-4 can achieve effective medical ICD-10 code normalization (assignment) by solely using the official WHO-supplied ICD-10 code descriptions. The findings suggest that the combination of GPT-4 with solely code descriptions might not be sufficient.

Relying solely on the WHO descriptions for medical coding can be problematic. A prime example is the challenge in coding “lower limb weakness” when it must fit into a broad category description like “Other malaise”, highlighting issues with semantic differences. Although the embedding methods used address some similarity issues, using an alphabetic index or leveraging training data might be more effective and accurate, but care must be taken to maintain generalizability.

A major challenge in using GPT-4 for research lies in its cost, processing speed, and privacy concerns. For each individual medical text, processing with GPT-4 using the proposed prompts typically takes several minutes at a minimum. Furthermore, when evaluating 50 code candidates, each accompanied by descriptions for the lead term extracted, the token use becomes significant, resulting in substantial costs. Additionally, GPT-4’s closed-source nature presents further complications in research applications.

Another limitation is the exclusive use of the CodiEsp dataset, which restricts the generalizability of our findings. While the MIMIC dataset is a commonly used public resource for ICD coding, it lacks annotations for explainability. No other publicly available ICD dataset is currently coded for explainability.

### Future Research

For NER of lead terms and their normalization to ICD codes, we suggest continuing with the NER approach for extracting lead terms, given its promising performance and its seamless integration into the workflow of medical coders. For future research, we propose implementing an agent-based LLM approach that mimics all steps of the medical coders’ workflow in detail.

Another point for future research is to explore the use of locally runnable LLMs, ensuring that data can remain in-house for privacy and security purposes.

### Conclusions

A dataset of ICD-10 lead terms, “CodiEsp-X-lead”, was created using few-shot prompting on GPT-4. This method of model-assisted labeling showcases its utility for other use cases where manual annotation is unfeasible, but reviewing the generated annotations is feasible. As GPT models are not the SOTA in NER [31], a RoBERTa-based model was fine-tuned, achieving an *F*_1_-score of 0.80. This model can aid medical coders in the primary step of medical coding by identifying ICD lead terms. GPT-4 was employed to generate code descriptions based on textual evidence, improving RAG retrieval for code lookups in a code description database. However, exclusive reliance on code descriptions, coupled with GPT-4 prompting for ICD-10 coding, led to only mediocre outcomes. Continuing with the NER approach for lead term extraction is advisable. Relying solely on code descriptions, along with GPT-4 prompts for ICD-10 coding, led to mediocre outcomes, partly because some codes in the dataset lacked descriptions. Additionally, this approach is likely less effective than mimicking the complete workflow of medical coders, which could yield more accurate results. A key limitation of using RAG for medical coding is its requirement for full-textual evidence upfront, making it less flexible than methods that extract evidence during the lookup process using official coding resources and guidelines.

## Supplementary material

10.2196/60095 Multimedia Appendix 1 GPT-4 parametric ICD-10 knowledge.

## Abbreviations

BERT: Bidirectional Encoder Representations from Transformer
ICD: International Classification of Diseases
LLM: large language model
NEC: not elsewhere classified
NER: named entity recognition
NLP: natural language processing
NOS: not otherwise specified
RAG: retrieval-augmented generation
RoBERTa: Robustly Optimized BERT Pretraining Approach
SOTA: state-of-the-art
WHO: World Health Organization
