# Supplementary material for: Developing an ICD-10 Coding Assistant: Pilot Study Using RoBERTa and GPT-4 for Term Extraction and Description-Based Code Selection
Source: JMIR Form Res. 2025 Feb 11;9:e60095. doi: 10.2196/60095 (PMC11835781; doi:10.2196/60095)
Supplement: Multimedia Appendix 1 [file formative-v9-e60095-s001.docx]

# GPT-4 parametric ICD-10 knowledge

Out-of-the-Box GPT-4 ICD-10 Code Recall (Diagnoses Only)

| **Evaluation** | **Matched (%)** |
| --- | --- |
| **Generated description vs official description refer to the same code** | 52 |
| **Sub-classification correct** | 47 |
| **Category correct** | 87 |
| **Chapter correct** | 97 |

When GPT-4 was given the task to identify the ICD-10 codes for 100 randomly chosen official diagnosis descriptions, GPT-4 assigned the correct code 47% of the time. It accurately identified the correct category or chapter in 87% and 97% of cases, respectively. GPT-4 failed to predict procedure codes, instead generating codes formatted like diagnosis codes.
